# Helmet-Mounted Real-Time Toxic Gas Monitoring and Prevention System for Workers in Confined Places

**DOI:** 10.3390/s23031590

**Published:** 2023-02-01

**Authors:** Janani Priyanka Perumpally Rajakumar, Jae-ho Choi

**Affiliations:** ICT Integrated Safety Ocean Smart Cities Engineering Department, Dong-A University, S12-401, 550 Bungil 37, Nakdong-Daero, Saha-Gu, Busan 49315, Republic of Korea

**Keywords:** toxic gas detection, real-time gas monitoring, confined places, Alphasense gas sensors, alert system

## Abstract

Occupational health and safety hazards associated with confined places are mainly caused by exposure to toxic gases and oxygen deficiency. Lack of awareness, inappropriate monitoring, and improper evacuation methods can lead to worker fatalities. Although previous studies have attempted to develop systems to solve this issue, limited research is available on their application in confined places. In this study, a real-time helmet-mounted system was developed to monitor major toxic gases (methane (CH_4_), hydrogen sulfide (H_2_S), ammonia (NH_3_), and carbon monoxide (CO)), oxygen, temperature, and humidity. Workers outside and inside confined spaces receive alerts every second to immediately initiate the rescue operation in the event of a hazard. The test results of a confined environment (wastewater treatment unit) highlighted that concentrations of CH_4_ and H_2_S were predominant (13 ppm). Compared to normal atmosphere, CH_4_ concentration was 122- and 130-fold higher in the landfill and digestion tanks, respectively, while H_2_S was 36- and 19-fold higher in the primary and secondary clarifiers, respectively. The oxygen content (18.2%) and humidity (33%) were below the minimum required limits. This study will benefit future research to target appropriate toxic gas monitoring and alert workers by studying the existing issues and associated factors in confined places.

## 1. Introduction

The increasing fatality rate of construction workers in confined workplaces is becoming a growing concern. Confined places have been classified as limited or restricted for entry and exit [1], with only limited space for workers to perform drainage, cleaning, or other maintenance tasks. Workplace accidents occur for a variety of reasons, including exposure to harmful substances or environments, fire or explosion, physical risks such as (falls, slips, or trips), contact with objects or equipment, and engulfment [2,3]. A confined atmosphere is dangerous and different from the normal atmosphere as it contains various toxic gases, with low oxygen levels [4]. Toxic gas inhalation remains a major cause of injuries and deaths in confined workers.

Several previous studies have focused on confined place accidents and fatality rates. Naghavi et al. [5] found that 70.91% of fatalities in Iran (from 2006 to 2017) were due to hydrogen sulfide, nitrogen poisoning, and oxygen deficiency, with explosions, electrocutions, and poisoning outside the confined place causing the remaining fatalities. The United States National Institute for Occupational Safety and Health (NIOSH) data and Italian data reports that oxygen deficiency and toxic gas poisoning caused major deaths in confined spaces between 1985–2015 and 2001–2016, respectively [6]. According to the Occupational Safety and Health Administration (OSHA) and Mine Safety and Health Administration (MSHA) reports, asphyxiation and toxic gas inhalation were the primary reasons contributing to an accident fatality rate of 1.5. Selman et al. [2] identified that 92% of deaths in confined places were caused by exposure to harmful substances or toxic environments. Reports from the Korea Occupational Safety and Health Agency (KOSHA) highlighted that 82.4% of accidents in South Korea between 2013–2015 occurred by inhaling harmful gases and poor, or lack of, ventilation [7]. In summary, insufficient or lack of awareness of the danger of confined places, improper rescue measurements, non-wearing of personal protective equipment, and poor ventilation results in increased fatality rate in confined spaces.

Construction workers frequently encounter numerous harmful gases while performing activities in confined spaces [4,8,9]. Previous studies have shown that hydrogen sulfide (H_2_S), methane (CH_4_), carbon monoxide (CO), and ammonia (NH_3_) are the major gases to which workers are often exposed [10,11,12,13,14,15]. Hydrogen sulfide is an important toxic gas with a “rotten egg” odor in confined places, such as sewers, wastewater treatment units, manholes, and other drainage areas [10]. Ammonia also has a pungent smell and can be detected at a concentration of 5 ppm [16,17]. Carbon monoxide is a poisonous gas, known as a “silent killer”, with no odor [18], while methane is a flammable explosive gas. These gases are colorless and can be produced by organic matter decomposition. Exposure to and inhaling these hazardous gases can lead to short- and long-term health impairments in workers. Dangerous health effects can be observed from exposure to small amounts of hazardous gases over a long period or in higher amounts over a short period. The major health impacts among workers include headache, irritation to the eyes, throat, and lungs, dizziness, nausea, vomiting, coughing, chest pain, unconsciousness, convulsions, fainting or sudden collapse, and even death [16,19,20,21,22,23,24].

Thus, appropriate monitoring and preventive measures should be considered to reduce the health effects of toxic gas inhalation and resulting accidents in confined places. Previous studies developed systems to measure toxic gases and alert workers in various fields. Yang et al. [25] monitored real-time CO concentrations using a wireless sensor network (WSN). This system, developed with pyroelectric sensors, was comprehensively tested in a controlled atmosphere with humidity levels between 50–60% and temperatures between 28–29 °C, thus making it suitable for industrial applications while measuring only a single gas concentration. Similarly, Illahi et al. [26] developed a smart helmet for CO monitoring using an MQ-7 sensor. Lin et al. [27] also used MQ-7 sensors to measure CO levels in parking garages using integrated WSN and building information modeling (BIM) technologies. However, the limited selectivity and narrow range of temperature and humidity of MQ-series sensors can result in inaccurate data [28].

Sinha et al. [29] utilized the IoT-based ZigBee technique to detect gases such as carbon dioxide (CO_2_), oxygen (O_2_), CH_4_, CO, and nitrogen (N_2_) and monitored personnel safety in underground mines. Crucial working environment factors (such as temperature and humidity) were not considered by the system; in addition, the system was not designed for confined spaces. Kuhar et al. [30] used smoke sensors, a GPS module (HC12), and an LED to develop a smart helmet for application at construction sites, primarily focusing on supervision and time management. Using smoke sensors is not appreciated because they consume more power, are inaccurate, and require frequent maintenance.

Vijay Kumar et al. [31] created a smart toxic gas monitoring system for sewage workers, comprising MQ-series gas sensors for measuring gases, with workers alerted through a Wi-Fi module ESP8266 attached to the system. In addition to the drawbacks of MQ-series sensors, the ESP8266 Wi-Fi module has lower efficiency, battery utilization, operating life, and larger current consumption [32]. Revanth et al. [33] developed a similar system using Nodemcu and GSM modules. However, this system does not include atmospheric conditions, and electronic interference and bandwidth lag can occur when the GSM module is used. In addition, these studies have not properly illustrated the decision criteria for alerts based on standard gas levels. Therefore, accurate and efficient toxic gas monitoring and improvements in confined worker safety are required to reduce accidents in confined areas [2].

This study aimed to develop a helmet-mounted system that can monitor hazardous gases efficiently and precisely in confined spaces. Considering existing information and limitations [34,35], Alphasense gas sensors were employed to evaluate gas concentrations. The smart helmet measures real-time concentrations of poisonous gases and confined atmospheric conditions. A quick and safe rescue operation is achieved by immediately alerting the worker inside and outside the confined space, possibly limiting the probability of health impacts (both short-term and long-term) on construction workers. The current study aims can be summarized as follows:

1. Monitor the real-time concentrations of primary toxic gases in confined places, such as methane, hydrogen sulfide, ammonia, and carbon monoxide, using a smart helmet.
2. Measure environmental parameters such as oxygen level, temperature, and humidity.
3. Provide alerts to workers inside and outside the confined place based on decision criteria.
4. Testing the helmet-mounted system in a confined environment to gauge its working performance.

The remainder of this paper is organized as follows. Section 2 describes the materials and the methodology used in this study. Section 3 discusses the design of the helmet-mounted system and the test results obtained. Finally, Section 4 further discusses the results and provides recommendations for future studies.

## 2. Materials and Methods

**Figure 1:**
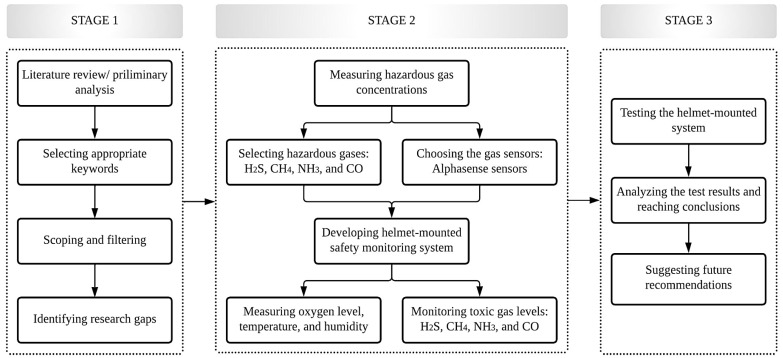
Overall study methodology.

The overall study methodology is illustrated in Figure 1. First, a preliminary analysis or literature review of previous studies was conducted, concentrating on hazardous gas exposure, health impacts of workers in confined places, toxic gas monitoring, and prevention systems to better understand existing knowledge and produce a system that overcomes the shortcomings of previous studies. Considering the focus of the journal and the selected keywords, literature from 1990–2022 was chosen. The keyword searching platforms were Science Direct, Web of Science, and Google Scholar; the keywords used were “toxic gas monitoring”, “confined places”, “toxic gas exposure”, “alert system”, “health impacts”, and “gas sensors”. Then, publications obtained from Google search results were filtered depending on the scope of the research. Studies related to hazardous gas poisoning in confined places, health impacts of workers involved in confined environments, and toxic gas monitoring methods in confined spaces were selected for the review process and extensively examined to evaluate the current status of toxic-gas monitoring and prevention studies. From this process, research gaps and future research directions were identified. In summary, the literature analysis clearly indicated that toxic gas exposure by workers in confined areas can negatively impact their health and safety.

**Figure 2:**
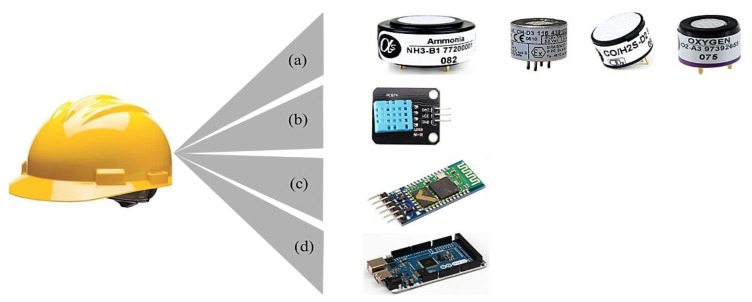
Components used to develop helmet-mounted monitoring system (**a**) Alphasense NH3-B1, CH-D3, CO/H2S-D2, O2-A3 gas sensors, (**b**) DHT11 temperature and humidity sensor, (**c**) HC-05 Bluetooth module, and (**d**) Arduino Mega.

After a literature review, the primary gases found in confined places were selected; H_2_S, CH_4_, NH_3_, and CO were among the most dangerous gases in confined areas. In parallel with the selection of gases, the choice of gas sensors is critical for designing a monitoring system. Electrochemical gas sensors developed by Alphasense Ltd. were employed in this study to enhance system effectiveness because of their accuracy, wide range of temperature and humidity, low power consumption, low cost, fast response, and regular calibration [34,35,36]. In addition to toxic gas levels, oxygen concentration levels were measured using an Alphasense oxygen gas sensor. Alphasense NH3-B1, CH-D3, and CO/H2S dual gas sensors and O2-A3 sensors were utilized to monitor the ammonia, methane, carbon monoxide, hydrogen sulfide, and oxygen concentrations. The atmospheric conditions in the confined workplace environment were measured using a DHT11 temperature and humidity sensor. The HC-05 Bluetooth module sent the recorded data and alerts to the worker outside the confined place. The entire system was mounted on a helmet, with the components or materials used illustrated in Figure 2.

The designed helmet-mounted safety system was tested under normal, toxic, and confined atmospheric conditions. The test results obtained were analyzed based on the standard exposure limits of the concerned authorities. Finally, the conclusions and future recommendations are provided.

## 3. Results

### 3.1. Design of the Helmet-Mounted System

**Figure 3:**
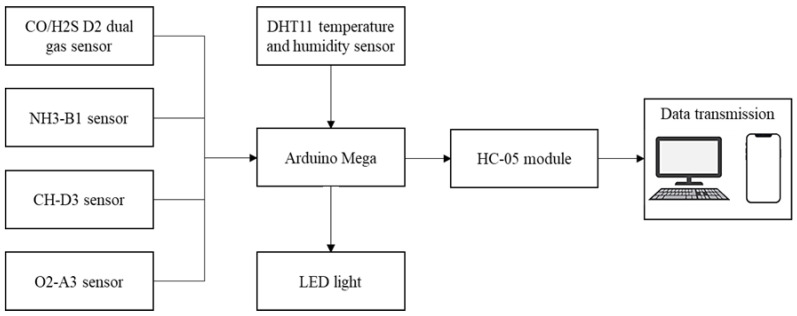
Block diagram of the system.

**Figure 4:**
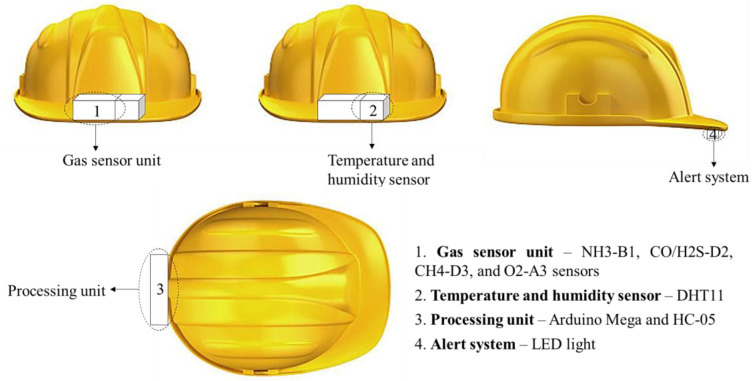
Positioning of toxic gas monitoring and prevention units on the helmet.

The effectiveness of a helmet-mounted system depends on the selection of the monitoring unit, which should be able to report real-time gas concentrations precisely and promptly to inform the worker. In this study, a helmet-mounted system was designed and developed to simultaneously measure the major toxic gases in confined places and the atmospheric conditions of the toxic gas environment. Figure 3 shows a block diagram of the proposed system. Alphasense gas sensors (such as CO/H2S, NH3-B1, CH-D3, and O2-A3), DHT11 temperature and humidity sensors, LED, and HC-05 Bluetooth module were connected to the Arduino Mega. An LED is used to provide real-time alerts to the worker inside the confined place, while the worker outside the confined place receives alerts and sensor readings via a mobile device or PC. The positioning of all units on the helmet is illustrated in Figure 4.

#### 3.1.1. Real-Time Gas Monitoring

Alphasense electrochemical gas sensors were used in this study to measure the gases. Several publications have advocated using these sensors for occupational and environmental gas monitoring. Penza et al. [36] used Alphasense sensors to monitor the concentrations of nitrogen dioxide (NO_2_), CO, sulfur dioxide (SO_2_), H_2_S, and particulate matter (PM_10_), suggesting that the sensor system is cost-effective and can be used for portable air quality monitoring. These sensors have the advantage of being effectively used in smart sensor networks in smart cities. Oletic and Bilas [37] measured the concentrations of CO, SO_2_, and NO_2_ using AlphaSense sensors. Using the analog front end (AFE), the output current from the sensor is converted to voltage; thus, the gas concentration is obtained from the output voltage. This study showed that the sensors were accurate within ±0.6 ppm CO and the power consumption was lower.

Afshar-Mohajer et al. [34] developed an air quality monitoring network for measuring ozone (O_3_), NO_2_, and CO and evaluated low-cost electrochemical sensors produced by Alphasense, Ltd. (Braintree, UK). A strong linear relationship was observed between the output voltages and gas concentrations (i.e., (R^2^ > 0.98). CO sensors were considered suitable for occupational and environmental CO monitoring because of their field-ready calibration capabilities. Marinov et al. [35] conducted a study that monitored NO_2_, O_3_, CO, and SO_2_ levels in the atmosphere using four-electrode amperometric gas sensors manufactured by Alphasense, with a four-sensor AFE board used for analog-to-digital conversion. These sensors were found to be applicable for low-power, low-cost, and fast-response air quality monitoring. The developed sensor interface was tested, and its accuracy was compared with that of the multichannel data acquisition (DAQ) system.

Sharma et al. [38] developed a low-cost sensor system by utilizing Alphasense electrochemical gas sensors to monitor NO_2_, O_3_, and PM_2.5_ levels in urban air. The findings of this study highlighted that the raw sensor data showed a determination coefficient (R^2^) of >0.80. The power consumption of the sensor system was under 0.5 W, leading to the authors recommending a sensor network for wireless systems in the field of air quality monitoring. Arroyo et al. [39] measured NO_2_, CO, O_3_, PM_2.5_, and PM_10_ concentrations in an outdoor environment. The R^2^ values obtained for the sensor data were in the range of 0.83–0.95, suggesting that the sensor system is suitable for portable air quality monitoring, with higher accuracy and noise reduction achievable by carefully designing the sensor interface.

**Figure 5:**
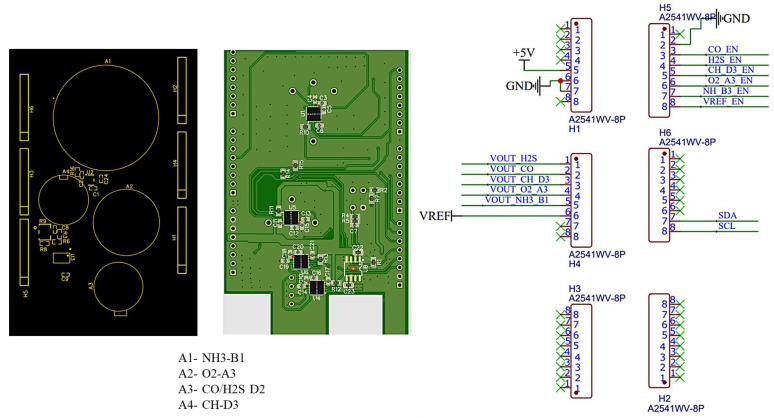
Designed gas sensor unit and connections with Arduino Mega.

In this study, a printed circuit board (PCB) was designed to combine gas sensors into a single unit. The output current from the Alphasense gas sensors was converted by employing an LMP91000 AFE, which helps signal communication between the sensor and microcontroller and converts the output cell current proportional to the output voltage. In addition, it supports programming with multiple electrochemical gas sensors and has a gas sensitivity range of 0.5–9500 nA/ppm, operating temperature between −40 °C to 85 °C, and voltage supply of 2.7–5.25 V. LMP91000 consumes a total current of 10μA and helps in noise reduction [40]. The developed sensor unit and connections of the PCB with the Arduino Mega are shown in Figure 5. The numbers 1 to 8 indicate the PINs of PCB for each header (i.e., H1, H2, H3, H4, H5, and H6).

The sensor unit was maintained inside a 3D mold and placed on the front side of the helmet. Workers were directly exposed to hazardous gases near their breathing zones. Regarding the safe level of breathing, sensors should be placed near the typical breathing zone, i.e., 4–6 feet from the ground [41]. Hence, the gas sensor unit was positioned at the front of the helmet to continuously and accurately measure the gas exposure concentrations.

#### 3.1.2. Temperature and Humidity Monitoring

The temperature and humidity of the confined environment are essential factors to be considered in the monitoring unit. The DHT11 temperature and humidity sensor was chosen for this purpose, with previous publications highly recommending using these sensors. The accuracy of the temperature and humidity measurements were ±2 °C and ±5% RH, respectively [42]. This sensor, comprising an 8-bit microcontroller, provides a calibrated digital output, precise temperature and humidity values, and responds quickly to exposure conditions. The DHT11 sensor has advantages such as low cost, simple interface, compactness, reliability, and stability [43,44,45]. In addition, Novelan and Amin [46], Saputro and Yantidewi [47], and Singh [48] utilized and suggested sensors for measuring temperature and humidity.

**Figure 6:**
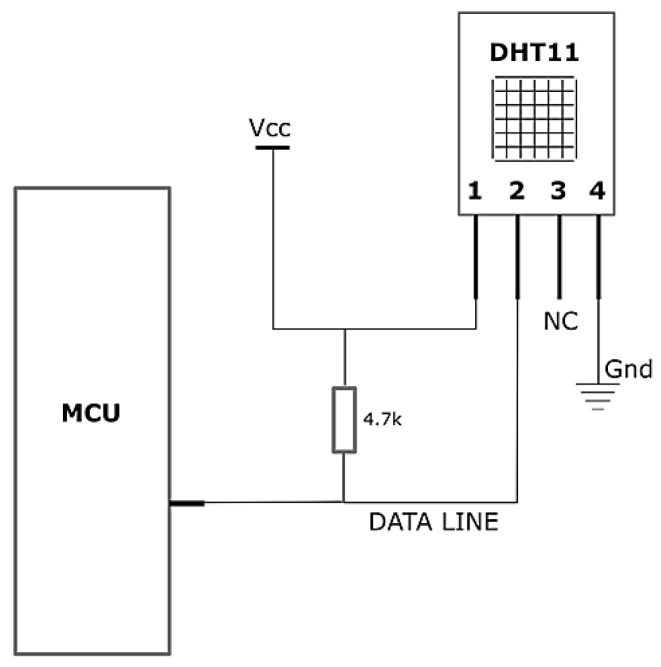
Connections of DHT11 with Arduino microprocessor (MCU).

Similar to the gas sensor unit, the DHT11 sensor was mounted on the front of the helmet near it because the atmospheric parameters experienced by workers must be measured simultaneously. Figure 6 shows the connections between the sensor and microprocessor (MCU). The PINs 1, 2, 3, and 4 indicate power input (VCC), data line, no connection (NC), and ground (Gnd), respectively.

#### 3.1.3. Wireless Communication

The exposure conditions and corresponding alert information must be relayed to the worker outside the confined place to avoid danger. For this purpose, the HC-05 Bluetooth module is utilized, which works on Serial Port Protocol (SPP) [49]. This module has a 2.4 GHz built-in antenna and operates between −25 to 75 °C. In addition, its compact size, easy connection with Bluetooth-enabled devices, better operating life, and low power consumption make it suitable for wireless communication.

**Figure 7:**
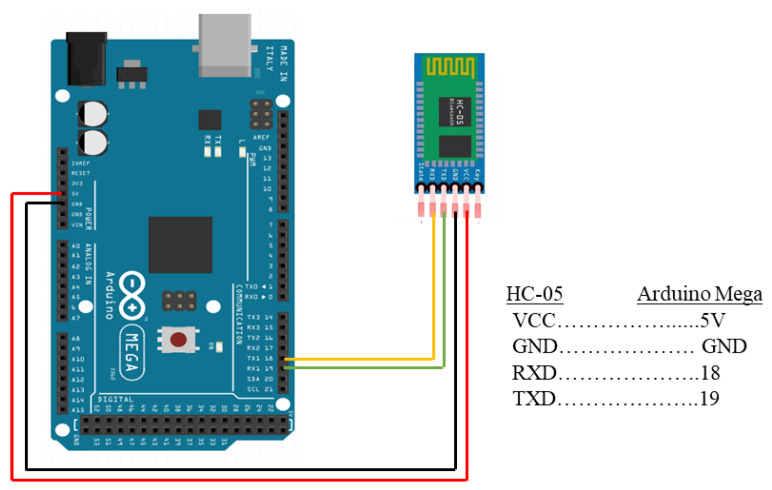
Connection diagram of HC-05 Bluetooth module with Arduino Mega.

Yadav and Vohra [32] analyzed the performance of an HC-05 Bluetooth module with an ESP8266 Wi-Fi module, with HC-05 exhibiting better battery utilization and efficiency than ESP8266. The Wi-Fi module used more current than HC-05, resulting in a shorter operating life. The modulation and transmit powers of HC-05 are 3 Mbps and +4 dBm RF, respectively [50]. The HC-05 module is cost-effective, sending signals quickly and with better quality [51]. The connection diagram of HC-05 with the Arduino Mega is illustrated in Figure 7. Power input or VCC, GND, transmit data or TXD, and receive data or RXD are represented by red, black, green, and yellow cables, respectively. 

#### 3.1.4. Alert System and Decision Criteria

**Figure 8:**
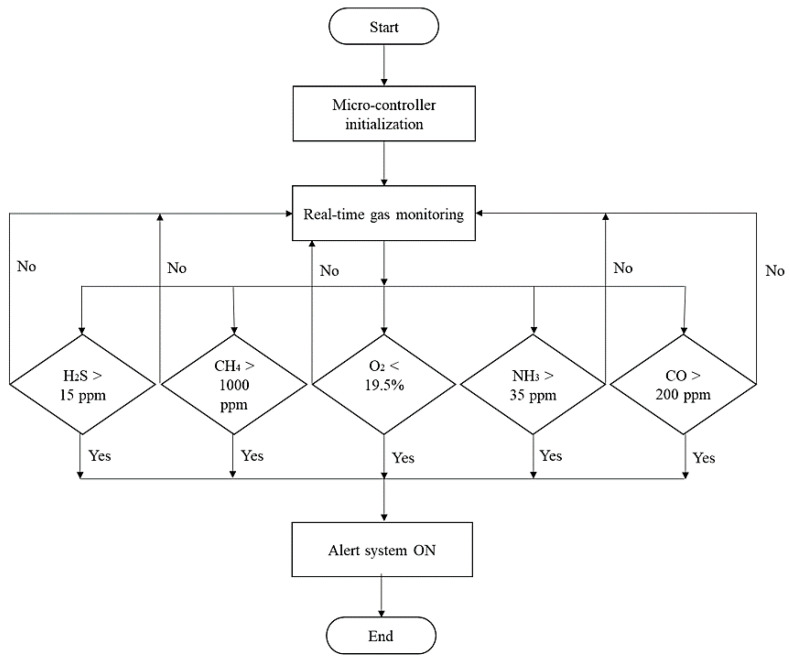
Decision criteria for the alert system.

An LED was provided at the front of the safety helmet to alert the worker inside the confined area. The decision criteria for the toxic gas exposure prevention system, based on the gas levels, are shown in Figure 8. The threshold limits of the gases are listed in Table 1 [52,53,54,55]. The short-term exposure limit (STEL) is the 15 min exposure that should not be increased at any time during the working day. The time-weighted average (TWA) refers to the maximum gas concentration for 8 h over a five-working-day week. Immediately Dangerous to Life or Health (IDLH) exposure standard is the peak concentration at which the exposure can be fatal if the worker is not equipped with sufficient respiratory protection units. In the current study, STEL was considered for the alert as real-time concentrations measured cannot be exceeded at that particular time. In the case of long-term monitoring, TWA can be utilized because it quantifies the mean exposure to the workers during a time period. For CH_4_, the maximum threshold limit is 1000 ppm during an 8 h working shift, as set by NIOSH. The alert system is activated when the toxic gas levels are higher and the oxygen concentration is lower than the recommended limits. If the levels are safe according to the standards, the alert system will be turned off, and the process will continue.

**Figure 9:**
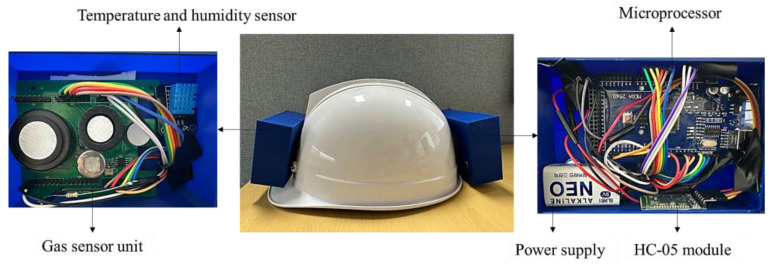
Developed helmet-mounted toxic gas monitoring and prevention system.

The developed helmet-mounted system is illustrated in Figure 9. The sensor system is portable, connected to a 9 V power supply and the Arduino can be powered for about 10 h to obtain the sensor data continuously. The data are transferred to the mobile device every second, and an alert is provided simultaneously. Therefore, rescue operations can be performed quickly to save workers’ lives without further hazardous gas exposure.

### 3.2. Testing of the Helmet-Mounted System

**Figure 10:**
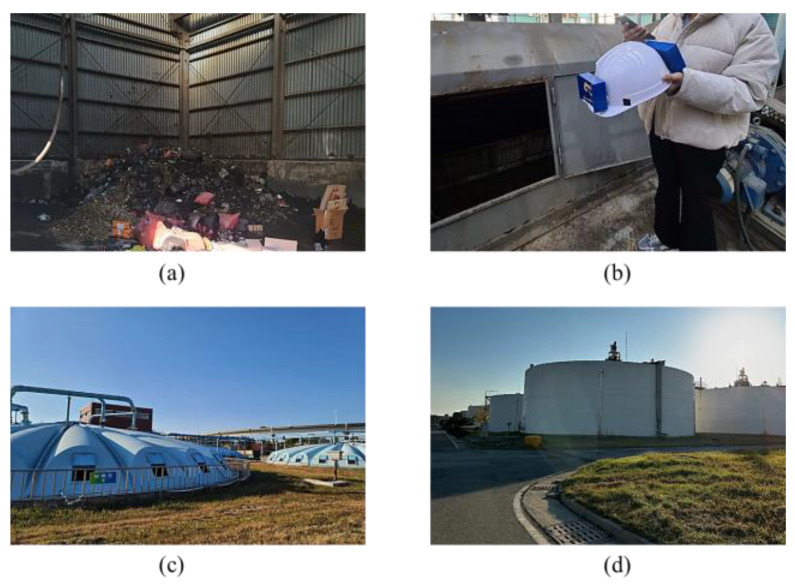
Data collection units in the wastewater treatment plant (**a**) landfill area, (**b**) primary sedimentation tank, (**c**) secondary clarifier, and (**d**) sludge digestion tank.

The helmet-mounted system was tested in normal and confined environments to compare the fluctuations in the sensor readings, with a wastewater treatment plant used for testing. The Jangrim wastewater treatment plant in Busan is a major municipal and industrial wastewater treatment plant in South Korea that collects and treats approximately 450 tons of wastewater. Figure 10 shows the units in the wastewater treatment plant where measurements were conducted—landfill area, primary sedimentation tank, secondary clarifier, and sludge digestion tank. Monitoring was performed for 10 min in both normal and toxic gas environments with a time interval of one second. If the atmospheric condition is dangerous, “Alert ON/danger” is sent to the mobile device, and safe conditions are displayed as “Alert OFF/Safe”. Therefore, workers outside the confined space can properly assess the situation accordingly.

**Figure 11:**
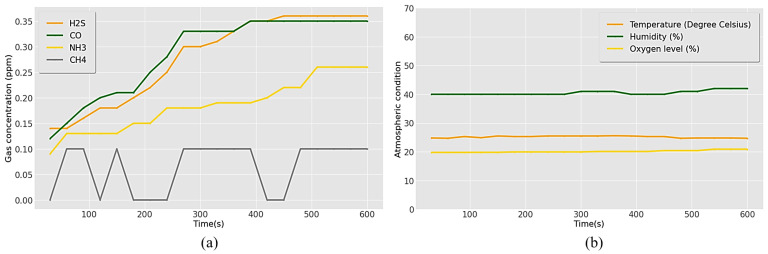
Recorded results for (**a**) concentration of toxic gases and (**b**) atmospheric parameters in the normal environment.

Table 2 and Figure 11 represent the data for the gases obtained and the exposure conditions in the normal atmosphere. The average and maximum concentrations recorded were 0.28 ppm and 0.36 ppm for H_2_S, 0.29 ppm and 0.35 ppm for CO, 0.19 ppm and 0.26 ppm for NH_3_, and 0.07 and 0.1 ppm for CH_4_ (Figure 11a). All toxic gas levels were under 1 ppm, considered safe according to the threshold limits. The mean humidity and temperature were 41% and 25.2 °C, with maximum levels of 42% and 25.6 °C, respectively. A humidity range of 40–70% is considered less hazardous to workers in confined places [56]. The optimal temperature for the working conditions was below 30 °C. Workplace temperatures of ≥30 °C can cause discomfort and heat stress. Hence, the temperature and humidity in a normal environment were found to be within these limits. An average oxygen concentration of 20.15% was obtained in a normal environment, higher than the recommended minimum level of 19.5% (Figure 11b).

**Figure 12:**
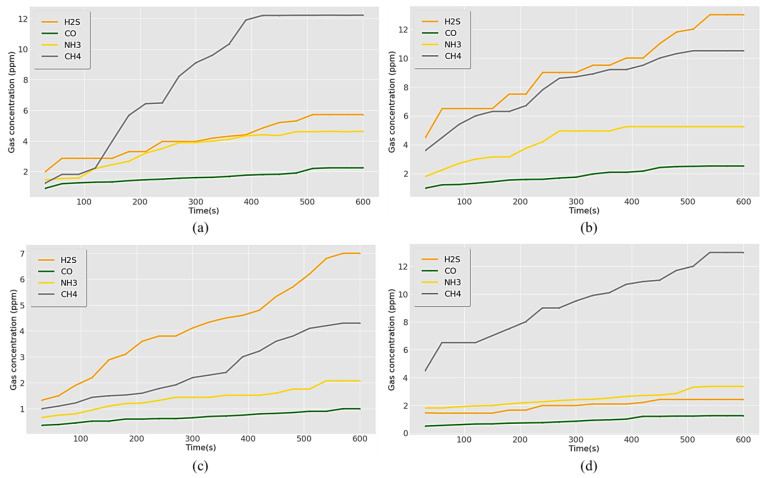
Recorded results for concentration of toxic gases in (**a**) landfill area, (**b**) primary sedimentation tank, (**c**) secondary clarifier, and (**d**) sludge digestion tank.

The recorded hazardous gas concentrations in various units of the wastewater treatment plant are shown in Figure 12. An increasing trend of gas concentrations was obtained, which reached a stable value after some time. The toxic gas data were initially inclined, with measurements taken for 10 min to obtain stable values. In a wastewater treatment plant, wastewater first undergoes a preliminary treatment where large objects, such as plastic, rags, and rubbish, are removed using special filter screens. These wastes were collected in a landfill area. A high concentration of CH_4_ was generated in the landfill area because of the natural bacterial decomposition of organic matter. The mean and maximum levels of CH_4_ were 8.21 ppm and 12.22 ppm, respectively. Followed by CH_4_, H_2_S showed mean and maximum exposure concentrations of 4.15 ppm and 5.72, while NH_3_ concentrations were 3.53 ppm and 4.62 ppm, respectively (Figure 12a).

After filtering, the wastewater is passed into the primary sedimentation tank, settling tank, or clarifier to remove the particles from the wastewater, and sludge is settled at the bottom of the tank. After primary sedimentation in the sedimentation tank, the floating sludge from the wastewater was collected in the secondary clarifier, and the total sludge is taken to the sludge treatment area. H_2_S exhibited the highest concentration in both units (Figure 12b,c). The average mean and maximum exposure values were 9.27 ppm and 13 ppm in the primary sedimentation tank and 4.22 ppm and 7 ppm in the secondary clarifier, respectively. The second-highest average and maximum levels were for CH_4,_ with values of 8.15 ppm and 10.5 ppm for the primary clarifier and 2.53 ppm and 4.3 ppm for the secondary clarifier. NH_3_ showed a maximum concentration of 5.25 ppm and 2.08 ppm for primary and secondary clarifiers, respectively (Figure 12b,c).

Sludge treatment was performed in digestion tanks where anaerobic digestion was performed by bacteria. CH_4_ was the primary gas generated during the anaerobic digestion process, similar to the results obtained for the landfill area. The mean and maximum concentrations of CH_4_ were 9.47 ppm and 13 ppm, respectively. For NH_3_ and H_2_S, the mean concentrations recorded were 2.5 ppm and 1.97 pm, and maximum levels were 3.36 ppm and 2.42 ppm, respectively (Figure 12d). CO concentrations were the lowest in all four monitored units. The maximum exposure values were 2.24 ppm, 2.52 ppm, 1, and 1.25 ppm in the landfill, primary sedimentation tank, secondary clarifiers, and digestion tanks, respectively. However, all the gas exposures were within the threshold limits set by the concerned authorities.

**Figure 13:**
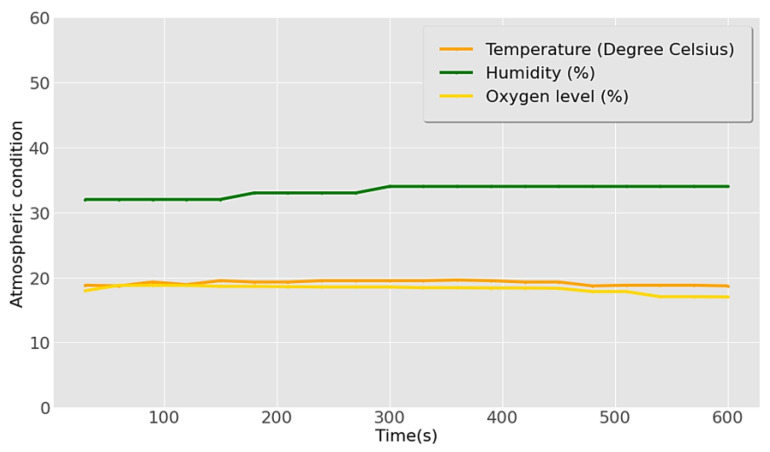
Results of atmospheric conditions in the wastewater treatment area.

Figure 13 shows the atmospheric conditions of the wastewater treatment and toxic gas environment. Mean values of 33% and 19.17 °C were determined for humidity and temperature, respectively, less than that in normal atmospheric conditions while the humidity was lower than the recommended value. The obtained mean oxygen level of 18.2% was lower than the minimum required level of 19.5%.

## 4. Discussion

The construction industry has been identified as a major source of workplace accidents and hazardous exposure fatalities. Physical hazards, toxic atmospheres, and engulfment can emerge as occupational health and safety risks. Hazardous gases are deadly and harmful when they enter confined atmospheres. Confined spaces are fully or partially enclosed areas in which only a limited number of workers can enter to perform assigned activities. Toxic gas accumulation, oxygen deficiency, poor ventilation, mechanical equipment usage, and flammable atmosphere can make confined atmospheres more dangerous than normal environments. Short-term and long-term exposure to hazardous gases creates various health issues, such as headaches, vomiting, unconsciousness, sudden collapses, and even death. Previous studies have highlighted that the hazards posed by toxic gases lead to the majority of confined place fatalities; thus, more attention is needed to properly mitigate this issue.

In this study, we developed a helmet-mounted toxic gas monitoring and prevention system that can measure the primary toxic gases in confined places such as H_2_S, NH_3_, CH_4_, and CO using Alphasense gas sensors; these sensors are suitable for low-cost, accurate, stable, fast response, low power, and portable occupational and environmental monitoring [34,35,36,37,38,39]. Oxygen level in air is also an important parameter to be considered in confined spaces. Alphasense CO/H2S D2, CH4-D3, NH3-B1, and O2-A3 sensors were employed for measuring concentrations of these gases, using a sensor unit designed and developed by the authors. Other exposure conditions, such as humidity and temperature, were also assessed using the DHT11 sensor. The monitoring unit was positioned at the front side of the helmet for measuring concentration levels near worker breathing zones every second. The toxic gas prevention system comprises an HC-05 Bluetooth module to provide real-time notifications to the worker or supervisor outside the confined location, while an LED alert is provided to the worker inside the confined location based on the developed decision criteria. As alerts are given within each second, rescue operations can be performed smoothly and quickly with proper usage of rescue equipment.

Testing was conducted in four units of the wastewater treatment plant: landfill, primary sedimentation tank, secondary clarifier, and digestion tank. A higher concentration of CH_4_ was recorded in the landfill area and sludge digestion tank, while the H_2_S content was higher in the primary and secondary clarifiers. The gases were mainly produced by the decomposition and anaerobic digestion processes. However, all gas levels were safe and below the threshold limits, with CO concentration the lowest in all the units. The maximum concentrations of CH_4_ in the landfill and digestion tanks were 122 and 130 times higher than the normal environment, respectively. In contrast, H_2_S levels were 36 and 19 times higher in the primary sedimentation tank and secondary clarifier, respectively. The oxygen level and humidity in the wastewater treatment units (18.2% and 33%, respectively) were lower than the recommended minimum limit. Thus, these results indicated fluctuations in gas concentrations and other exposure conditions of confined atmospheres compared with normal environments.

The current study included safety monitoring of toxic gas exposure for workers involved in confined spaces. The findings of this study will allow future researchers to broaden the safety monitoring system to a health and safety monitoring system by integrating workers’ health conditions such as pulse rate, blood oxygen level, body temperature, and EEG or brainwave analysis. The effects of humidity and temperature on sensor readings, comparison of sensor data with bench march devices, short- and long-term exposure assessments, sensor dynamics analysis, and displaying system status can enhance the performance and efficiency of the monitoring system. Combining toxic gas monitoring and the measurement of other major pollutants in the construction industry, such as particulate matter, can mitigate occupational health impacts at construction sites.

## Figures and Tables

**Table 1:** Threshold limits of gases.

Type of Gas	TWA (ppm)	STEL (ppm)	IDLH (ppm)
Hydrogen sulfide (H_2_S)	10	15	100
Carbon monoxide (CO)	30	200	1200
Ammonia (NH_3_)	25	35	300
Oxygen (O_2_)	19.5%		

**Table 2:** Result of data obtained from the helmet-mounted system in the normal environment.

Time (s)	Toxic Gas Concentration (ppm)				Temperature (°C)	Humidity (%)	Oxygen (%)
	H_2_S	CO	NH_3_	CH_4_			
30	0.14	0.12	0.09	0	24.8	40	19.78
60	0.14	0.15	0.13	0.10	24.7	40	19.78
90	0.16	0.18	0.13	0.10	25.3	40	19.78
120	0.18	0.20	0.13	0	24.9	40	19.80
150	0.18	0.21	0.13	0.10	25.5	40	19.80
180	0.20	0.21	0.15	0	25.3	40	19.95
210	0.22	0.25	0.15	0	25.3	40	19.95
240	0.25	0.28	0.18	0	25.5	40	19.95
270	0.30	0.33	0.18	0.10	25.5	40	19.95
300	0.30	0.33	0.18	0.10	25.5	41	19.95
330	0.31	0.33	0.19	0.10	25.5	41	20.10
360	0.33	0.33	0.19	0.10	25.6	41	20.10
390	0.35	0.35	0.19	0.10	25.5	40	20.10
420	0.35	0.35	0.20	0	25.3	40	20.10
450	0.36	0.35	0.22	0	25.3	40	20.40
480	0.36	0.35	0.22	0.10	24.7	41	20.40
510	0.36	0.35	0.26	0.10	24.8	41	20.40
540	0.36	0.35	0.26	0.10	24.8	42	20.90
570	0.36	0.35	0.26	0.10	24.8	42	20.90
600	0.36	0.35	0.26	0.10	24.7	42	20.90

## Data Availability

All relevant data are within the manuscript.
